# Self-perceived substance and behavioral addictions among Jewish Israeli adolescents during the COVID-19 pandemic

**DOI:** 10.1016/j.abrep.2022.100431

**Published:** 2022-05-11

**Authors:** Yaniv Efrati, Marcantonio M. Spada

**Affiliations:** a Bar-Ilan University, Faculty of Education, Ramat Gan, Israel; b Division of Psychology, School of Applied Sciences, London South Bank University, London, UK

**Keywords:** Adolescents, Behavioral addictions, COVID-19, Self-perceived, Sociodemographic categories, Substance use disorder

## Abstract

This study examined self-perceived substance and behavioral addictions among Israeli adolescents during the COVID-19 pandemic across different sociodemographic categories. The sample comprised 2,074 adolescents (40% males, 60% females) aged 12–19 years who completed the survey anonymously and with parental consent. We examined what is the prevalence of self-perceived substance and behavioral addictions in this population in the COVID-19 pandemic context. Participants reported self-perceived addictions to social networks (70%), shopping (46%), binge eating (34%), gaming (30%), sex-related behavior (15%), psychoactive substance (31%, including alcohol, tobacco, cannabis, and/or cocaine), and gambling (3%). Moreover, differences were found to be directly related to age, biological sex, religiosity, socioeconomic status, and immigration status. From a lay epidemiological perspective, the current research expands our knowledge about self-perceived addiction among adolescents during the COVID-19 pandemic, offering better understanding of the likelihood probability factors for self-perceived addiction among adolescents and its related negative outcomes, including increased risk factors for later adult life.

## Highlights

• Overall rates found in self-perceived substance and behavioral addictions among Israeli adolescents during the COVID-19 pandemic.
• Differences found in self-perceived substance and behavioral addictions by age, biological sex, religiosity, immigration status, and socioeconomic status.

## 1 Introduction

Adolescence is associated with high levels of risk-taking (Ciranka & van den Bos, 2021) with a peak in risky behaviors, such as substance and behavioral addictions (Van Rooij et al., 2014). Substance and behavioral addictions are defined by functional impairment in daily life, increasing priority given to (and preoccupation with) substance use or specific behavior, and continuation or escalation of substance use or specific behavior despite the occurrence of negative consequences (Brand et al., 2020, Zou et al., 2017). To date, much of the research on substance and behavioral addictions among adolescents has focused on frequency and use behavior (see the Health Behaviour in School-aged Children study [HBSC]; Walsh et al., 2020). However, few studies have provided a broader view of self-perceived substance and behavioral addictions among adolescents. In this research, we adopt a lay epidemiological approach that considers the self-perceived of adolescents from the general population in Israel. We also examine common warning signs and symptoms of problematic substance abuse and behavioral addiction.

Lay epidemiology proposes that “fields of symptomatology, nosology, aetiology, and epidemiology have identifiable counterparts in the thoughts and activities of people outside the formal medical community” (Davison, Smith, & Frankel, 1991, p. 6). From this perspective, the lay public’s conceptions of addiction can have important implications regarding whether or not an individual identifies themselves as an addict (Hodgins et al., 2022, Schluter et al., 2020). Adolescents who use the term “sense of self” perceive their addiction as an extension of themselves because it reflects their sense of self-identity, which is linked to how they want to present themselves to others (e.g., Jameel, Shahnawaz, & Griffiths, 2019). Wild et al. (2015) suggest that prevalence rates of self-attributed addiction problems exhibit a striking concordance with those obtained using expert-derived, formal diagnostic criteria. Moreover, epidemiological studies can lead or support the interventions for addictive behaviors (Rumpf et al., 2019). Therefore, the first aim of the current study was to examine adolescents’ self-perceived substance and behavioral addiction.

Addiction among adolescents can be discussed using two levels of language: “social language” and “mental health language.” Social language is commonly used in society and in online media; it is often guilty of overpathologizing everyday life experiences (Billieux et al., 2015). Using social language, young persons can explore the theme of self and identify themselves or others as being in trouble and in need of eliminating addictive behavior. The problem is that “social language” can be used by an individual to describe themselves as an addict when objective clinical measures contradict this. Conversely, an adolescent may not recognize that they have a clinically defined addiction, and this mistaken perception may lead to failure to take responsibility using an external rather than internal control focus. In addition, adolescence is a period of development, and it might be an appropriate time to detect disposition to addiction. “mental health language” is employed in discussions on “addiction” in research and in the setting of clinical criteria for the professional diagnosis and treatment of addiction. However, even if we speak of addiction in terms of “social” or “mental health language”, the present study sheds further light on the self-perceived subjective aspects of addiction in the framework of a lay epidemiological approach, which can have clinical implications.

In addition to this timely question, we also wanted to focus specifically on exploring substance and behavioral addictions during the COVID-19 pandemic. Recent studies have found increased rates of addiction since the outbreak of the coronavirus among adults and adolescents: drugs (Cisneros and Cunningham, 2021, Cisneros and Cunningham, 2021, Mallet et al., 2021, Mallet et al., 2021, Nguyen and Buxton, 2021, Slavova et al., 2020, Slavova et al., 2020); alcohol (Blithikioti et al., 2021, Blithikioti et al., 2021, Kar et al., 2021, Kar et al., 2021); tobacco/vaping (Yang and Ma, 2021, Yang and Ma, 2021); marijuana/cannabis (Levy et al., 2021, Levy et al., 2021); the internet (Islam et al., 2020, Király et al., 2020, Oka et al., 2021); gaming (López-Cabarcos et al., 2020, López-Cabarcos et al., 2020); gambling (Brodeur et al., 2021, Wardle et al., 2021); shopping (Koch et al., 2020, Moon et al., 2021); binge eating (Schulte et al., 2022, Trott et al., 2021); pornography (Grubbs et al., 2021, Sallie et al., 2021); sex-related behavior (Döring, 2020, Lehmiller et al., 2021), and social networks (Gómez-Galán et al., 2020, Gómez-Galán et al., 2020, Luo et al., 2021, Luo et al., 2021). A recent study involving Israeli adolescents indicates that 37% of young people aged 12–18 have had to self-isolate since the pandemic began and 28.9% have been confined to their homes in a lockdown context more than once (Gewirtz-Meydan et al., 2021). Social distancing measures implemented to curtail the spread of COVID-19 have had a negative impact on the mental health of young people, who have shown higher rates of behavioral addiction enabled by the internet (gaming, social media, etc.).

Research indicates that the average age of first substance use is 13–14, which applies mainly to alcohol and illicit substances (Nowak et al., 2018, Nowak et al., 2018). Moreover, previous studies have demonstrated that substance use before 16–18 is associated with later substance abuse (Scholes-Balog et al., 2016, Scholes-Balog et al., 2016, Jordan and Andersen, 2017, Jordan and Andersen, 2017, Rioux et al., 2018, Rioux et al., 2018, Brumback et al., 2021). Substance use and attitudes have changed over the decades, with reports indicating an increase in problematic consequences or a higher prevalence of addiction among young adults and adults (Deputy et al., 2021, Deputy et al., 2021). For example, in a U.S.-based review, Carliner, Brown, Sarvet, & Hasin (2017) found that since the early 2000 s, adult and adolescent perception of cannabis use as risky has decreased, while over the same period, the prevalence of cannabis use among adolescents has not changed significantly. However, adult cannabis use, disorders, and related consequences have increased. In the context of pornography use in adolescents, Farré and colleagues (2020) found that prevalence increases with older ages. Our hypothesis is that older adolescents will report a higher level of substance addiction and sex-related behavior, whereas there will not be any differences based on age for other types of behavioral addiction (gaming, social networks, etc.).

Gender plays a key role in substance and behavioral addictions. For example, substance use disorders are more prevalent in males than in females (McHugh et al., 2018, McHugh et al., 2018). In a survey conducted by the Center for Behavioral Health Statistics and Quality (Center for Behavioral Health Statistics and Quality, 2016), an estimated 47.4% of females aged 12 and older reported past-month alcohol use, compared to 56.2% of males in the same age group. Females also displayed lower rates of binge drinking, heavy drinking, and alcohol use disorder than males. Nicotine dependence was present in 52.3% of female habitual smokers and 9.7% of females overall. In the same report, 7.9% of females and 12.5% of males aged 12 and older reported past-month illicit drug use; corresponding rates of illicit drug use disorders stood at 2% and 3.8% (Center for Behavioral Health Statistics and Quality, 2016). In Norway, cannabis use among university students met the criteria for cannabis use disorder in 6% of the population (3.8% females; 8.5% males) (Andreas et al., 2021). Gender differences are also common in behavioral addictions. For example, research indicates that male adolescents are exposed to pornography at an earlier age than females, watch more pornography, and more often tend to describe themselves as being addicted to pornography (Kowalewska et al., 2020, Peter and Valkenburg, 2016). In addition, males in this age group show greater attentional bias toward sexual cues and a higher prevalence of compulsive sexual behavior disorder (Efrati & Amichai-Hamburger, 2021). With regards to gambling, the prevalence of gambling disorder in young people aged between 11 and 16 years was estimated at 4.6% (Calado et al., 2017, Montiel et al., 2021), and more frequent in men than in women in the adult group (Ronzitti et al., 2016). Similarly, studies on gaming addiction have shown a higher prevalence in males than in females (Efrati, Kolubinski, Marino, & Spada, 2021). Conversely, some studies suggest that more females prefer to use the internet for communication, while males prefer game playing (Mihara & Higuchi, 2017), and social networking addiction has been shown to be more prevalent among adolescent females than males (Peris, de la Barrera, Schoeps, & Montoya-Castilla, 2020). In a similar vein, a meta-analysis of adult-representative studies, showed a pooled prevalence of shopping addiction in different populations of approximately 5%, with women exhibiting higher percentages compared to men, a prevalence that has been increasing over the years (Maraz et al., 2016). Finally, Rodrigue, Gearhardt, and Begin (2019) have shown that binge eating is nearly as prevalent in young people as in adults. In Turkey, a study of 612 high school students revealed that 12.4% had food addictions, with females found to have a higher food addiction score than males (Candan & Küçük, 2019). Accordingly, our second hypothesis is that the prevalence of self-perceived substance use, gambling, gaming, and sex-related addictions will be higher among male adolescents than among females of the same age. On the other hand, we predict that more females will report having addictions to shopping, binge–eating, and social networks than males.

Religious affiliation belief seems to be another factor that influences addictive behavioral patterns. Research indicates that religion is often seen as a buffer or barrier against risk behavior and substance and behavioral addictions (see Connery and Devido, 2020, Grubbs and Grant, 2020). Religious adolescents and adults are less likely to experience substance addictions (Acheampong et al., 2016, Grim and Grim, 2019, Miller et al., 2000). In some aspects of life, however, religious belief may cause an inner struggle that might sustain an addictive behavior (Faigin, Pargament, & Abu-Raiya, 2014). According to the moral incongruence model (Grubbs et al., 2020, Grubbs et al., 2020, Lewczuk et al., 2020, Grubbs et al., 2019) emotional and physical distress arises from the contradiction between a person’s moral beliefs and the behavior in which they engage. For example, there is incongruence between the natural sexual urges of a religious adolescent and the conservative principles endorsed by their religious leaders (e.g., a rabbi) and foundational literature, such as the Bible or the Talmud, in which sexual thoughts and behaviors are discouraged or even condemned. Consistent with this incongruence, a recent study on Polish adults with self-perceived behavioral addiction to pornography, internet use, social networking, or online gaming found that religiosity was uniquely, although weakly, connected to pornography addiction, but not to other types of addictive behaviors (Lewczuk et al., 2021). Therefore, our third hypothesis is that secular adolescents will report more self-perceived substance and behavioral addiction.

Regarding socioeconomic status (SES), research indicates that there are associations between socioeconomic factors and substance abuse outcomes (Collins, 2016, Diala et al., 2004, Melchior et al., 2007). For example, previous research on tobacco found that low SES was associated with a higher prevalence and co-occurrence of risk factors (Wellman et al., 2018). Moreover, Petruzelka et al. (2020) found an association between socioeconomic status and substance and behavioral addiction (risky internet use, gambling) in adolescents. In their research on sex-related behavior, Efrati & Amichai-Hamburger (2021) found that adolescents who engage in both offline and online sexual activities have a lower economic status, on average, than those without sexual experience. Therefore, we hypothesize that adolescents with a low SES will report more self-perceived substance and behavioral addiction.

Finally, with regard to immigration, research has indicated that young people from families with a more vulnerable immigrant status are at risk of immigration-related adversities and behavioral withdrawal, which in turn lead to difficulties of adjustment among adolescents and high substance use (Roche et al., 2021). In addition, among immigrant adolescents, there are more severe levels of problem gambling and a higher distribution of at-risk and problem gamblers with respect to non-immigrant adolescents (Donati, Primi, Mazzarese, Sanson, & Leone, 2020). Therefore, our final hypothesis is that having an immigrant background is likely to increase the likelihood of self-perceived substance and behavioral addiction among adolescents.

In the current study, we administered a survey to investigate self-perceived substance addiction (alcohol, tobacco, cannabis, and cocaine) and behavioral addiction (shopping, gaming, gambling, binge eating, sexual activity, and social networks) among Jewish adolescents in Israel, the largest population group in the country (74%) (Central Bureau of Statistics, Jerusalem, Israel, 2020). This is the first time that large-scale research in this specific area has been conducted in Israel. We hypothesized that there would be noticeable differences based on the sociodemographic variables of age, gender, religiosity, SES, and immigration status focusing in particular on religiosity due to the largely discrete levels of Jewish practice and identification in Israel that allow for easy categorization and comparison (Pew Research Center, 2016).

## 2 Method

### 2.1 Participants

The study population comprised 2,074 Jewish Israeli adolescents from the general community (825 males and 1,249 females), aged 12–19 (M = 16.14, SD = 1.35), all enrolled in the eighth (n = 94), ninth (n = 317), tenth (n = 464), eleventh (n = 490), and twelfth (n = 682) grades (missing report; [n = 27]). Most (96%) were native Israelis. Socioeconomically, 0.3% of participants described their level as being very bad, 3.8% bad, 61.7% good, and 33.9% very good. In terms of religious affiliation, the sample consisted of 997 (48.1%) self-reported religious individuals, of which 391 (18.9%) traditional, 587 (28.3%) secular, and 99 (4.8%) ultra-Orthodox.

### 2.2 Measures

**Sociodemographic variables**. Adolescents reported their age group (12–14, 15–17, 18–19 years), biological sex (male, female), religiosity (secular, traditional, religious, ultra-Orthodox), immigration status (Israeli, immigrant), and socioeconomic status (or SES, divided into the categories of very good, good, bad, and very bad).

**The Screener for Substance and Behavioral Addictions (SSBA)** (Schluter et al., 2018). The SSBA is a brief screening instrument for measuring self-attributed addiction problems in the general population. It is comprised of four self-report items, each reflecting a distinct sign or symptom of potentially problematic involvement (“I did it too much;” “Once I started, I couldn’t stop;” “I felt I had to do it in order to function;” and “I continued to do it, even though it caused problems”), administered for each of four substances (alcohol, tobacco, cannabis, and cocaine), and six behaviors (gambling, shopping, videogaming, eating, sexual activity, and working [because the focus is on adolescents, in the present study, I adapted the questionnaire to “social networking” instead of “working”]). Participants were asked to rate each item in terms of frequency in the previous 12 months on a 5-point Likert scale: 0 = none of the time, 1 = not much of the time, 2 = some of the time, 3 = most of the time, and 4 = all of the time. Two additional response options were available: “I didn’t do this at all” and “Don’t know/I prefer not to say.” Participants were also provided with brief definitions of each behavior. To reduce the risk that participants would misunderstand what types of problems the questions were meant to address, descriptions of excessive behavior were provided explaining the symptoms of certain disorders (namely related to food and sex). We calculated an average score for the four questions; participants who obtained an average score of 2 or higher for a self-perceived addiction were defined as having an addiction. The coefficient alpha was 0.85.

### 2.3 Procedure

The study was presented to participants as a research project on addiction in Jewish adolescents from various regions of Israel (males and females, secular and religious, from the eastern, central, southern, or northern parts of Israel). The participants constituted a convenience sample recruited from a variety of sources (postings on bulletin boards and in online forums). Questionnaires were uploaded to Qualtrics, an online platform for questionnaires, and distributed by several research assistants. Parents of adolescents who agreed to participate in the study were contacted via email and/or phone and were asked to review the questionnaires and sign an informed parental consent form, which was sent back to the research assistants by email. Upon agreement, a link to the online survey was sent to the participant who was assured anonymity. Participants were then asked to complete the survey in private, in a quiet room in their home (without the presence of others). Following receipt of a signed informed consent form, questionnaires were presented in random order. All questionnaires were in Hebrew, Israel’s the native language. Lastly, there was an online debriefing and participants were thanked for their participation. The analysis was conducted during the COVID-19 pandemic from March 2021 to July 2021 in Israel. The procedure was approved by the Institutional Review Board (IRB).

### 2.4 Data analysis

Rates of self-perceived substance and behavioral addiction among Israeli adolescents – alcohol, tobacco, cannabis, cocaine, gambling, shopping, gaming, binge eating, sex-related behavior, and social networking – were reported accompanied by Blaker’s exact 95% confidence intervals (CIs). Differences in these rates by age (12–14, 15–17, 18–19), biological sex (males, females), religiosity (secular, traditional, religious, ultra-Orthodox), immigration status (Israeli, immigrant), and socioeconomic status (SES: very good, good, bad, very bad) were determined by chi-square tests for independence of measures and odds ratios with 95% CIs. For religiosity, the secular group was compared with the other groups when calculating the odds ratios, and for SES, the “very good” status group was compared with the other groups.

## 3 Results

### 3.1 Overall rates of self-perceived substance and behavioral addiction among Israeli adolescents during the COVID-19 pandemic

**Fig. 1:**
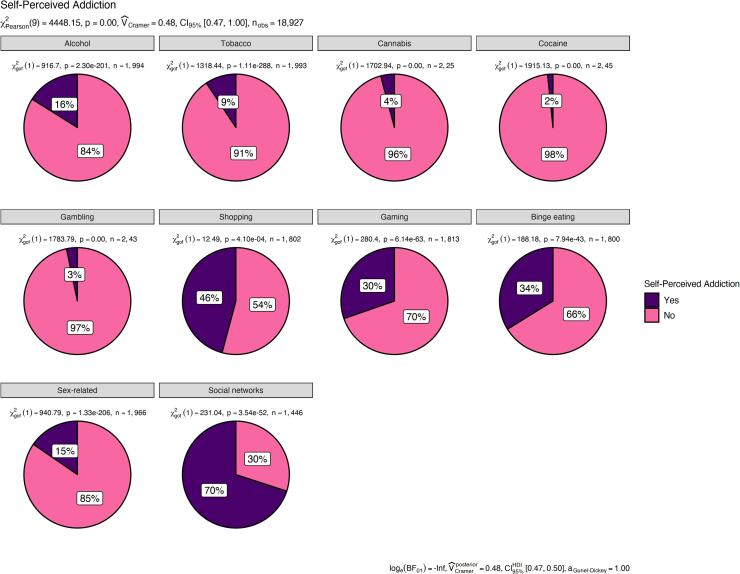
Rates of self-perceived addictive behaviors among Israeli adolescents during the COVID-19 pandemic.

Overall rates are presented in Fig. 1. Results indicated that 31% of Israeli adolescents identified themselves as having an addiction to psychoactive substance (alcohol, tobacco, cannabis, and/or cocaine) during the COVID-19 pandemic with 46% stating that they had a shopping addiction, 34% reporting that they suffered from binge eating, and 30% reporting a gaming addiction. In addition, 15% stated that they had sex-related addictions, whereas 70% acknowledged an addiction to a social networking. Finally, 3% reported a gambling addiction.

**Fig. 2:**
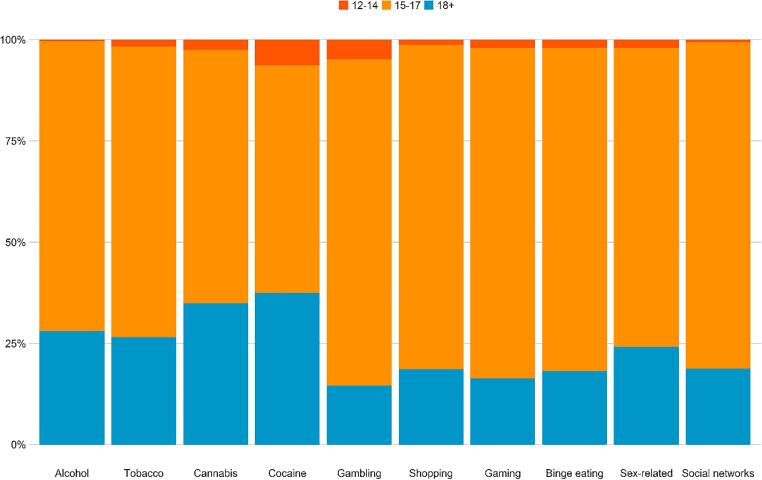
Differences in the rates of self-perceived addictive behaviors by age groups (values are calculated as a percentage of those who identified themselves as addicts in each domain).

**Differences related to age.** Age-related differences are presented in Fig. 2 and Table 1. The analyses indicated that adolescents in the 15–17 age group were 49% and 54% less likely to have alcohol and tobacco addictions, respectively, than those in the 18–19 age group. Twelve-to-fourteen-year-olds were 87% less likely to have a self-attributed alcohol addiction. In addition, those in the 15–17 age group were 31% less likely to have a sex-related addiction than the 18–19 age group.

**Table 1:** Rates of self-perceived addictive behaviors among Israeli adolescents as a function of age groups.

	18+		15–17			12–14		
	*n*	*% (95% CI)*	*n*	*% (95% CI)*	*OR (95% CI)*	*n*	*% (95% CI)*	*OR (95% CI)*
Alcohol	84	0.25 (0.20, 0.30)	234	0.14 (0.13, 0.16)	0.51 (0.39, 0.68) ***	1	0.04 (0.00, 0.19)	0.13 (0.00, 0.80) *
Tobacco	53	0.16 (0.12, 0.20)	130	0.08 (0.07, 0.09)	0.46 (0.32, 0.66) ***	2	0.08 (0.02, 0.26)	0.48 (0.05, 2.06)
Cannabis	21	0.06 (0.04, 0.09)	60	0.04 (0.03, 0.05)	0.58 (0.34, 1.03)	2	0.08 (0.02, 0.26)	1.41 (0.15, 6.40)
Cocaine	9	0.03 (0.01, 0.05)	20	0.01 (0.01, 0.02)	0.47 (0.20, 1.18)	3	0.12 (0.03, 0.30)	5.18 (0.84, 22.81)
Gambling	13	0.04 (0.02, 0.06)	51	0.03 (0.02, 0.04)	0.82 (0.43, 1.67)	2	0.08 (0.02, 0.26)	2.36 (0.24, 11.47)
Shopping	141	0.44 (0.39, 0.50)	673	0.46 (0.44, 0.49)	1.07 (0.83, 1.38)	10	0.45 (0.26, 0.67)	1.04 (0.39, 2.71)
Gaming	83	0.26 (0.21, 0.31)	455	0.31 (0.29, 0.34)	1.31 (0.99, 1.74)	10	0.43 (0.23, 0.63)	2.22 (0.84, 5.71)
Binge eating	111	0.35 (0.30, 0.40)	486	0.33 (0.31, 0.36)	0.94 (0.72, 1.22)	10	0.42 (0.23, 0.63)	1.33 (0.51, 3.34)
Sex-related	66	0.20 (0.16, 0.24)	232	0.14 (0.13, 0.16)	0.69 (0.51, 0.95) *	4	0.17 (0.06, 0.37)	0.82 (0.20, 2.56)
Social networks	171	0.66 (0.60, 0.71)	829	0.71 (0.69, 0.74)	1.30 (0.96, 1.74)	10	0.50 (0.29, 0.71)	0.52 (0.19, 1.45)

Note. 95% CI = 95% confidence intervals. OR = odds ratio. * *p* <.05, ** *p* <.01, *** *p* <.001. The 18 + group served as the reference group for the ORs.

**Fig. 3:**
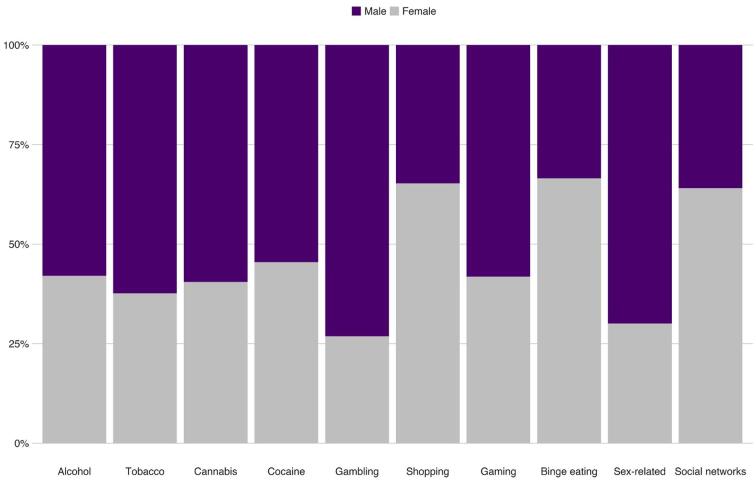
Differences in the rates of self-perceived addictive behaviors by biological sex (values are calculated as a percentage of those who identified themselves as addicts in each domain).

**Fig. 4:**
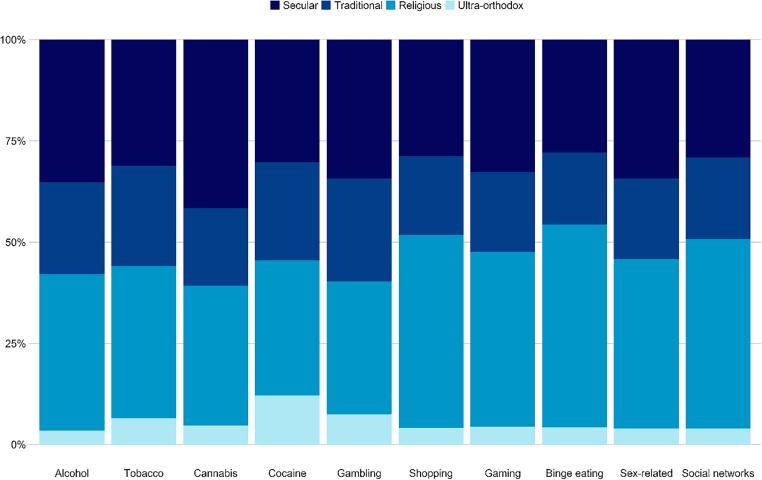
Differences in the rates of self-reported addictive behaviors by religiosity (values are calculated as a percentage of those who identified themselves as addicts in each domain).

**Differences related to biological sex.** Sex differences are presented in Fig. 3 and Table 2. The analyses indicated that female adolescents were less likely than males to identify themselves as having addictions to alcohol (69% less), tobacco (66% less), cannabis (47% less), gambling (77% less), gaming (73% less) and sex-related behavior (79% less). Females were more likely to report addictions to shopping (59% more likely), binge eating (59% more), and social networks (137% more) than were males (see Fig. 4).

**Table 2:** Rates of self-perceived addictive behaviors among Israeli adolescents as a function of biological sex.

	Males		Females		
	*n*	*% (95% CI)*	*n*	*% (95% CI)*	*OR (95% CI)*
Alcohol	186	0.24 (0.21, 0.27)	135	0.11 (0.09, 0.13)	0.39 (0.31, 0.51) ***
Tobacco	116	0.15 (0.13, 0.18)	70	0.06 (0.05, 0.07)	0.34 (0.25, 0.47) ***
Cannabis	50	0.06 (0.05, 0.08)	34	0.03 (0.02, 0.04)	0.43 (0.26, 0.68) ***
Cocaine	18	0.02 (0.01, 0.03)	15	0.01 (0.01, 0.02)	0.53 (0.25, 1.14)
Gambling	49	0.06 (0.05, 0.08)	18	0.01 (0.01, 0.02)	0.23 (0.12, 0.40) ***
Shopping	287	0.39 (0.36, 0.43)	539	0.5 (0.47, 0.53)	1.59 (1.31, 1,93) ***
Gaming	320	0.48 (0.44, 0.52)	230	0.20 (0.18, 0.22)	0.27 (0.22, 0.33) ***
Binge eating	204	0.28 (0.25, 0.31)	405	0.38 (0.35, 0.41)	1.59 (1.29, 1.96) ***
Sex-related	212	0.28 (0.25, 0.31)	91	0.08 (0.06, 0.09)	0.21 (0.16, 0.27) ***
Social networks	364	0.59 (0.55, 0.63)	648	0.78 (0.75, 0.80)	2.37 (1.87, 3.00) ***

Note. 95% CI = 95% confidence intervals. OR = odds ratio. * *p* <.05, ** *p* <.01, *** *p* <.001.

**Fig. 5:**
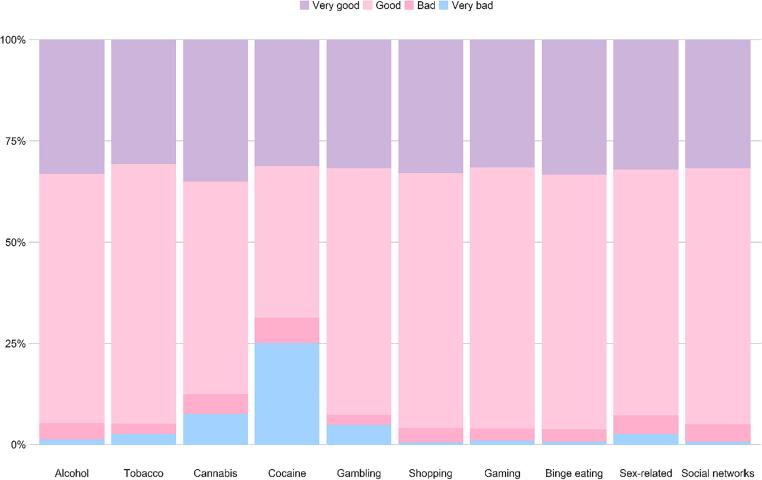
Differences in the rates of self-reported addictive behaviors by SES (values are calculated as a percentage of those who identified themselves as addicts in each domain).

**Differences related to religiosity.** Differences based on religiosity are presented in Fig. 5 and Table 3. The analyses indicated that religious individuals from the sample were less likely than those adhering to the secular culture to identify themselves as having addictions to alcohol (42% less), tobacco (34% less), cannabis (53% less), gaming (37% less), and sex-related activity (36% less). Ultra-Orthodox adolescents were 65% less likely than the secular category to perceive themselves as having an addiction to social networks.

**Table 3:** Rates of self-perceived addictive behaviors among Israeli adolescents as a function of religiosity.

	Secular		Traditional			Religious			Ultra-Orthodox		
	*n*	*% (95% CI)*	*n*	*% (95% CI)*	*OR (95% CI)*	*n*	*% (95% CI)*	*OR (95% CI)*	*n*	*% (95% CI)*	*OR (95% CI)*
Alcohol	113	0.20 (0.17, 0.24)	73	0.20 (0.16, 0.24)	0.99 (0.70, 1.39)	124	0.13 (0.11, 0.15)	0.58 (0.43, 0.78) ***	11	0.12 (0.06, 0.20)	0.53 (0.25, 1.05)
Tobacco	58	0.11 (0.08, 0.13)	46	0.12 (0.9, 0.16)	1.19 (0.77, 1.83)	70	0.07 (0.06, 0.09)	0.66 (0.45, 0.96) *	12	0.12 (0.07, 0.20)	1.19 (0.56, 2.37)
Cannabis	35	0.06 (0.04, 0.08)	16	0.04 (0.3, 0.7)	0.67 (0.34, 1.27)	29	0.03 (0.02, 0.04)	0.47 (0.27, 0.80) **	4	0.04 (0.01, 0.10)	0.68 (0.17, 1.96)
Cocaine	10	0.02 (0.01, 0.03)	8	0.02 (0.01, 0.04)	1.21 (0.41, 3,43)	11	0.01 (0.01, 0.02)	0.64 (0.25, 1.70)	4	0.04 (0.01, 0.10)	2.47 (0.55, 8.79)
Gambling	23	0.04 (0.03, 0.06)	17	0.04 (0.03, 0.07)	1.13 (0.56, 2.24)	22	0.02 (0.01, 0.03)	0.55 (0.29, 1.04)	5	0.05 (0.02, 0.12)	1.33 (0.38, 3.69)
Shopping	238	0.48 (0.44, 0.53)	160	0.48 (0.42, 0.53)	0.98 (0.73, 1.30)	394	0.44 (0.41, 0.48)	0.86 (0.68, 1.07)	34	0.41 (0.31, 0.52)	0.75 (0.45, 1.22)
Gaming	180	0.36 (0.32, 0.41)	108	0.32 (0.27, 0.38)	0.84 (0.62, 1.13)	238	0.27 (0.24, 0.30)	0.63 (0.50, 0.81) ***	24	0.28 (0.19, 0.38)	0.67 (0.38, 1.13)
Binge eating	170	0.33 (0.29, 0.37)	108	0.34 (0.29, 0.39)	1.04 (0.76, 1.41)	305	0.35 (0.32, 0.38)	1.09 (0.86, 1.39)	26	0.31 (0.21, 0.41)	0.90 (0.53, 1.51)
Sex-related	104	0.19 (0.16, 0.23)	60	0.16 (0.12, 0.20)	0.81 (0.56, 1.16)	127	0.13 (0.11, 0.15)	0.64 (0.48, 0.86) **	12	0.13 (0.07, 0.22)	0.66 (0.31, 1.27)
Social networks	295	0.74 (0.69, 0.78)	204	0.75 (0.69, 0.80)	1.05 (0.73, 1.52)	473	0.68 (0.65, 0.72)	0.77 (0.58, 1.02)	40	0.49 (0.38, 0.61)	0.35 (0.21, 0.58) ***

Note. 95% CI = 95% confidence intervals. OR = odds ratio. * *p* <.05, ** *p* <.01, *** *p* <.001. The secular group served as the reference group for the ORs.

**Differences related to SES.** Differences relating to SES are presented in Fig. 5 and Table 4. The analyses indicated that adolescents with a “very bad” SES were more likely to report having addictions to alcohol (by 617%), tobacco (by 1,636%), cannabis (by 2,212%), gambling (by 7,772%) and sex-related activities (by 1,269%) than adolescents in the “very good” SES category. Adolescents with a “good” or “bad” SES were also more likely to identify themselves as having sex-related addictions (by 34% and 104%, respectively) than adolescents in the “very good” SES group. Finally, adolescents in the “good” SES category were also more likely to report binge eating (by 26%) and social networks addiction (by 44%) than adolescents in the “very good” SES group.

**Table 4:** Rates of self-perceived addictive behaviors among Israeli adolescents as a function of SES.

	Very good		Good			Bad			Very bad		
	*n*	*% (95% CI)*	*n*	*% (95% CI)*	*OR (95% CI)*	*n*	*% (95% CI)*	*OR (95% CI)*	*n*	*% (95% CI)*	*OR (95% CI)*
Alcohol	105	0.16 (0.13, 0.19)	195	0.16 (0.14, 0.18)	1.01 (0.78, 1.33)	16	0.22 (0.13, 0.32)	1.49 (0.77, 2.75)	4	0.57 (0.23, 0.87)	7.17 (1.19, 49.69) *
Tobacco	53	0.08 (0.06, 0.10)	122	0.10 (0.08, 0.12)	1.28 (0.90, 1.83)	7	0.10 (0.05, 0.19)	1.30 (0.48, 3.04)	3	0.60 (0.19, 0.92)	17.36 (1.95, 211.73) **
Cannabis	28	0.04 (0.03, 0.06)	47	0.04 (0.03, 0.05)	0.92 (0.56, 1.54)	5	0.07 (0.03, 0.15)	1.68 (0.49, 4.60)	3	0.50 (0.15, 0.85)	23.12 (2.96, 180.71) **
Cocaine	11	0.02 (0.01, 0.03)	14	0.01 (0.01, 0.02)	0.69 (0.29, 1.69)	4	0.05 (0.02, 0.12)	3.32 (0.75, 11.60)	4	0.57 (0.23, 0.87)	78.72 (11.86, 593.10) ***
Gambling	21	0.03 (0.02, 0.05)	40	0.03 (0.02, 0.04)	1.03 (0.59, 1.86)	2	0.03 (0.00, 0.09)	0.86 (0.10, 3.62)	4	0.67 (0.27, 0.94)	61.57 (8.31, 732.23) ***
Shopping	267	0.44 (0.40, 0.48)	516	0.46 (0.43, 0.49)	1.09 (0.98, 1.34)	35	0.51 (0.39, 0.62)	1.30 (0.77, 2.21)	4	0.67 (0.27, 0.94)	2.53 (0.36, 28.15)
Gaming	178	0.29 (0.25, 0.32)	346	0.31 (0.28, 0.34)	1.11 (0.89, 1.39)	19	0.29 (0.19, 0.41)	1.02 (0.55, 1.84)	6	1.00 (0.59, 1.00)	Inf (2.87, Inf)
Binge eating	191	0.31 (0.27, 0.34)	393	0.36 (0.33, 0.39)	1.26 (1.01, 1.56) *	20	0.33 (0.22, 0.47)	1.14 (0.61, 2.05)	4	0.67 (0.27, 0.94)	4.53 (0.64, 0.50.49)
Sex-related	84	0.13 (0.10, 0.15)	199	0.16 (0.14, 0.19)	1.34 (1.01, 1.79) *	16	0.23 (0.14, 0.34)	2.04 (1.04, 3.82) *	4	0.67 (0.27, 0.94)	13.69 (1.93, 153.40) **
Social networks	315	0.65 (0.60, 0.69)	643	0.72 (0.69, 0.75)	1.44 (1.12, 1.83) **	44	0.77 (0.64, 0.87)	1.86 (0.95, 3.87)	6	0.86 (0.45, 0.99)	3.29 (0.39, 152.34)

Note. 95% CI = 95% confidence intervals. OR = odds ratio. * *p* <.05, ** *p* <.01, *** *p* <.001. The “very good” SES group served as the reference group for the ORs.

**Fig. 6:**
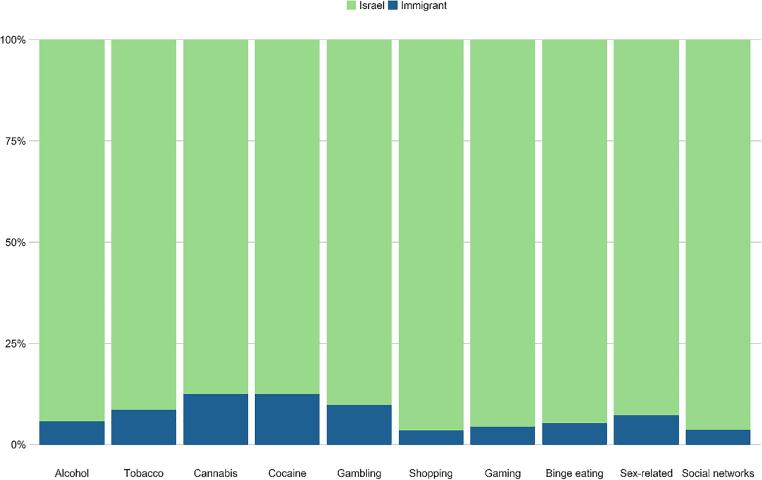
Differences in the rates of self-reported addictive behaviors by immigration (values are calculated as a percentage of those who identified themselves as addicts in each domain).

**Differences related to immigration status.** Differences relating to immigration status are presented in Fig. 6 and Table 5. The analyses indicated that immigrant adolescents were more likely to identify themselves as having alcohol (by 105%), tobacco (by 150%), cannabis (by 289%), and gambling (by 268%) addictions than native born Israelis.

**Table 5:** Rates of self-perceived addictive behaviors among Israeli adolescents as a function of immigration.

	Israel		Other		
	*n*	*% (95% CI)*	*n*	*% (95% CI)*	*OR (95% CI)*
Alcohol	300	0.16 (0.14, 0.17)	21	0.28 (0.18, 0.39)	2.05 (1.16, 3.51) **
Tobacco	171	0.09 (0.08, 0.10)	15	0.20 (0.12, 0.30)	2.50 (1.29, 4.58) **
Cannabis	74	0.04 (0.03, 0.05)	10	0.13 (0.07, 0.23)	3.89 (1.71, 8.01) ***
Cocaine	29	0.01 (0.01, 0.02)	4	0.05 (0.02, 0.13)	3.70 (0.92, 10.95)
Gambling	59	0.03 (0.02, 0.04)	8	0.10 (0.05, 0.19)	3.68 (1.46, 8.14) **
Shopping	795	0.46 (0.44, 0.48)	30	0.43 (0.31, 0.55)	0.88 (0.52, 1.46)
Gaming	526	0.30 (0.28, 0.32)	22	0.34 (0.23, 0.47)	1.21 (0.68, 2.10)
Binge eating	576	0.33 (0.31, 0.36)	32	0.45 (0.34, 0.57)	1.64 (0.98, 2.71)
Sex-related	287	0.15 (0.14, 0.17)	16	0.22 (0.13, 0.33)	1.57 (0.83, 2.81)
Social networks	967	0.70 (0.68, 0.72)	42	0.67 (0.54, 0.77)	0.85 (0.49, 1.53)

Note. 95% CI = 95% confidence intervals. OR = odds ratio. * *p* <.05, ** *p* <.01, *** *p* <.001.

## 4 Discussion

The current study highlights self-perceived substance and behavioral addiction as a key factor that could contribute to prevention and disclosure of addiction among adolescents in Israel. In this research, we focused on two questions: (a) What is the prevalence of self-perceived substance and behavioral addictions in this population in the COVID-19 pandemic context? (b) Are there differences relating to age, biological sex, religiosity, immigration status, and SES? To this end, we conducted a large-scale study involving 2,074 Jewish Israeli adolescents from the general population.

In keeping with the study’s hypotheses, 31% of adolescents reported an addiction to a psychoactive substance (alcohol, tobacco, cannabis, and/or cocaine). Consistent with studies on the effects of substance use among adults during the COVID-19 pandemic (Nguyen & Buxton, 2021), participants who acknowledged self-perceived substance addiction also reported increased use in riskier settings (taking drugs alone, higher consumption, or stockpiling of drugs); worsening mental health; covert and overt school dropout; increased risk of relapse after a period of abstinence; reduced access to both school counseling and social services; access to an increasingly toxic supply of drugs; and reduced tolerance when at risk of relapse and a return to regular use. Education, welfare, and healthcare policies should pay attention to these components when dealing with adolescents substance abuse. In addition, we found that participants reported a high prevalence of self-perceived addiction related to the use of screens and technology: social networks (70%), shopping (46%), gaming (34%), and sex-related behavior (15%). These levels of prevalence can be explained by the COVID-19 context. Indeed, it has been shown that the use of screen technologies has approximately doubled among children and teenagers since before the onset of the pandemic (Li et al., 2021, Ophir et al., 2022) and adolescents were confined to their homes for at least one lockdown period (Gewirtz-Meydan et al., 2021), triggering feelings of isolation. According to recent studies, the highest increase in screen use has been for the purposes of entertainment among young children and adolescents (Götz et al., 2020, Ophir et al., 2022, Schmidt et al., 2020). Moreover, with lockdown periods forcing young people to spend increased amounts of time at home, it is not surprising that the adolescents assessed in this study reported high of binge eating (30%), since food and opportunities to eat became more accessible, and so-called “comfort eating” can be a way of compensating for boredom or distress.

Unsurprisingly, and in keeping with the hypotheses, differences were found relating to age: older adolescents display higher rates of tobacco and alcohol addiction. In addition, the same age group was more likely to acknowledge sex-related behavior. Previous research has also shown that older adolescents report higher levels of addiction to substance use and pornography (Carliner et al., 2017, Farré et al., 2020). This finding may be explained by greater exposure to alcohol, tobacco, and sex-related behavior, all of which are more accessible in the context of social events attended by teenagers, reflecting social acceptance.

In general, males reported a higher prevalence of substance and behavioral addiction than females based on a wide range of studies on adolescent populations (Schulte et al., 2009). As we hypothesized, in this research, higher percentages of male participants identified themselves as having alcohol, tobacco, cannabis, gambling, gaming, and sex-related addictions. Females, however, were more likely than males to describe themselves as having addictions to shopping, binge eating, and social networks. These findings correspond with previous studies on adults indicating higher compulsive buying behavior scores among women (Maraz, Griffiths, & Demetrovics, 2016) and more severe dependence on cultural mechanisms among individuals living in developed countries (Black, Monahan, Schlosser, & Repertinger, 2001). Since Israel is categorized as a developed country, food can be used as a luxury, and more females report binge eating than males, because physiological development changes in the adolescent female body can result in preoccupations with “body image” and issues with food. The problem is compounded by the cultural image of the “beauty model” which puts teenage females under constant pressure to look a certain way, and may even lead to eating disorders. Finally, the COVID-19 pandemic has intensified the emotional and physical stress resulting from overeating as a form of compensation or escapism. Another major form of escapism during the COVID-19 pandemic has been social networking. Social networks are readily available and accessible and during lockdown periods provided a way of creating interpersonal connections and a forum for emotional support. Previous research studies have already demonstrated that females prefer to use the internet for communication purposes (Mihara & Higuchi, 2017), which can explain why a recent study in Israel found that female adolescents report more severe social network addiction than males (Efrati et al., 2021).

The population of Israel is composed of different types of religious, traditional, modern, and ultra-Orthodox groups. Confirming our hypothesis, non-religious individuals (adhering to secular principles) showed a higher tendency to identify themselves as having an addiction to alcohol, tobacco, cannabis, gaming, or sex-related behavior. Previous studies have suggested that religion acts as a buffer or deterrent against patterns of substance use (Connery & Devido, 2020) and gaming (Lewczuk et al., 2021). To account for this finding, adolescents do not need to self-define themselves as addicted in order to be troubled by thoughts of sexual behaviors. In this study, the term “self-perceived” addiction allows for an individual’s recognition of the problem, and previous studies have shown that religious adolescents (Efrati, 2019, Efrati et al., 2021) suppress sexual thoughts and therefore report compulsive sexual behavior and psychopathology (Efrati & Dannon, 2018). Ultra-Orthodox adolescents were less likely than secular adolescents to identify themselves as having an addiction to social networks. In ultra-Orthodox society, geographically concentrated in certain dense residential neighborhoods, children and adolescents are educated in institutions that are under constant supervision, and the experience of mobility and social networking is very limited (Rosenberg, Blondheim, & Katz, 2019).

Regarding SES, the results indicated that, confirming the hypothesis, adolescents with a “very bad” SES were more likely to report problems with alcohol, tobacco, cannabis, gambling, and sex-related behavior. Previous research found that there is an increased risk of substance use among younger residents in both disadvantaged and middle-class neighborhoods (Karriker-Jaffe, 2013). This study proposes that environmental interventions targeting disadvantaged neighborhoods may help prevent substance use, gambling, and sex-related behavior. In particular, in lower-class neighborhoods, there are gaps in adolescents education regarding the issues of substance use, gambling, and sex-related behavior.

Finally, we found that immigrant adolescents were more likely to self-report perceived alcohol, tobacco, cannabis, and gambling addictions than native born Israelis. This finding corresponds with other studies which revealed high substance use among immigrant adolescents (Roche et al., 2021) and high levels of gambling (Donati et al., 2020). Thus, these results support the culturally informed psychological theory that recognizes immigrant generational status as a social position variable i.e., a factor placing individuals in a social hierarchy, which can set developmental risk processes in motion (García Coll et al., 1996, Stein et al., 2016).

## 5 Limitations and future studies

The results of the current study should be considered in light of its limitations. The study was based on self-report measures, which may have been subject to response bias. This is especially relevant for items that address intimate subjects, such as addiction behavior. Because the design was cross-sectional, causal relations between the study variables could not be inferred. Longitudinal studies are necessary to determine the directionality of the associations between psychological, cognitive, psychopathological, and cultural characteristics in self-reported addiction. Finally, the research population was comprised of Jewish adolescents from the general population of Israel. Future studies should examine various other ethnic and cultural populations to ascertain the replicability and generalizability of the findings.

To date, studies in Israel have examined the prevalence of substance and behavioral addiction (the HBSC; Walsh et al., 2020). This study represents the first examination in Israel of self-perceived addiction among adolescents, especially during the COVID-19 pandemic. Unfortunately, a comparison before and during the COVID-19 pandemic is not possible, but future studies may examine self-perceived substance and behavioral addiction after the COVID-19 pandemic.

Despite these limitations, the current study has revealed patterns of self-perceived substance and behavioral addiction among Israeli adolescents. These tendencies are, without doubt, related to the “normal,” adventurous, and psychological developments that characterize adolescence. On the other hand, they might also help us to gain a better understanding of the likelihood probability factors for self-perceived addiction among adolescents and its related negative outcomes, including increased risk factors for later adult life.

A review of the literature of the past decades offers strong support for addiction prevention programs among adolescents (Nation et al., 2003, Throuvala et al., 2019). Our findings are indicative of the importance of approaches that address a broad definition of addiction and focus on well-being, affirmation and inclusivity in tackling adolescents' addiction. Notably, they reinforce the guidance to schools provided by the Israeli Ministry of Education on prevention addiction standards and further strengthen the justification for their widespread adoption. The implications of this work for school communities are important. In short, attention to the full range of self-perceived substance and behavioral addiction topics, scaffolded across grades, embedded in supportive school environments and across subject areas, has the potential to improve social, and emotional health, and academic outcomes for young people.

## 6 Consent

All information was recorded anonymously, and respondents were assured that personal information would be kept confidential.

## Ethical approval

The Institutional Review Board (IRB) determined this study was exempt from requiring IRB approval.

## Declaration of Competing Interest

The authors declare that they have no known competing financial interests or personal relationships that could have appeared to influence the work reported in this paper. Given their role as Editor Spada M. had no involvement in the peer-review of this article and had no access to information regarding its peer-review.
